# The emerging potential of the psycho-cardiovascular interaction network in precision medicine

**DOI:** 10.1093/pcmedi/pbag026

**Published:** 2026-09-12

**Authors:** Jiayu Liao

**Affiliations:** Department of Bioengineering, Bourns College of Engineering, University of California at Riverside, Riverside, CA 92521, United States; Biomedical Science, School of Medicine, University of California at Riverside, Riverside, CA 92521, United States

Cardiovascular medicine has traditionally emphasized lipids, blood pressure, glycemic status, and cardiac structure, while psychological factors have often been treated as secondary consequences of disease. That separation is increasingly untenable. Chronic stress, depression, anxiety, and other psychiatric conditions may precede cardiovascular disease (CVD), alter sleep, physical activity, medication adherence, and health-care engagement, and intensify after cardiovascular events. These exposures can converge on autonomic, neuroendocrine, endothelial, metabolic, thrombotic, and inflammatory pathways, creating reciprocal trajectories between mental and cardiovascular health [1, 2].

Against this background, the Review by Li and colleagues introduces the psycho-cardiovascular interaction network (PCIN), a framework intended to connect psychological and psychiatric states with cardiovascular phenotypes across epidemiological, physiological, cellular, and molecular levels [3]. The authors extend the broader brain–heart axis concept by organizing the field around clinically recognizable psychological exposures; intermediate mechanisms, including hypothalamic-pituitary-adrenal axis and autonomic imbalance, chronic inflammation, platelet activation, unhealthy behaviors, and sleep or circadian disruption; and five representative signaling systems: BDNF, PROK/PKR, MAPK, 5-HT/5-HT_2_A-R, and the NLRP3 inflammasome. This organization is timely because it places behavioral, physiological, and molecular observations within a common translational framework rather than treating them as unrelated correlates.

The principal conceptual value of PCIN is therefore its insistence on multilevel integration. The framework encourages investigators to link a defined psychological phenotype to an intermediate physiological process, a molecular mediator, and a cardiovascular endpoint. It also accommodates bidirectionality: psychological distress may promote cardiovascular injury, whereas myocardial infarction, heart failure, arrhythmia, and other cardiac conditions may generate afferent neural, immune, hemodynamic, and behavioral signals that worsen mental health. Recent neurocardiology studies similarly implicate autonomic circuits and neuroimmune communication involving bone marrow and spleen, while emphasizing that translation from experimental models to human disease remains incomplete [4–6].

Nevertheless, the word “network” establishes a demanding scientific standard. At present, PCIN is best regarded as a hypothesis-generating framework rather than a validated biological or clinical network. A predictive network requires defined nodes and edges, directionality, temporal ordering, context-dependent weights, feedback loops, and measurable outputs. The current model does not yet establish whether the proposed pathways operate sequentially, in parallel, or only within specific patient subgroups. Psychological-to-cardiovascular and cardiovascular-to-psychological pathways should be modeled separately before their feedback structure is integrated. Dynamic systems approaches, longitudinal sampling, mediation analyses, and perturbation studies will be needed to determine which connections are causal, redundant, compensatory, or clinically dominant.

The five signaling systems illustrate both the strength and the present limits of the framework. BDNF/TrkB signaling plausibly connects synaptic plasticity, endothelial survival, angiogenesis, nitric oxide signaling, and myocardial calcium handling; PROK/PKR signaling links neurogenesis and circadian or olfactory biology with vascular development and myocardial repair; MAPK signaling provides a broad stress-responsive kinase architecture; 5-HT/5-HT_2_A-R signaling intersects neurotransmission, platelet activation, vascular tone, and cardiac remodeling; and NLRP3 offers a compelling inflammatory bridge among chronic stress, neuroinflammation, myocardial injury, fibrosis, and pyroptosis [3]. However, participation of a pathway in both brain and cardiovascular tissues does not by itself demonstrate cross-organ mediation. Isoform, receptor, cell-type, compartment, dose, and disease-stage effects may even be directionally opposed. Human studies must therefore establish temporal mediation and tissue-relevant target engagement rather than infer a brain–heart mechanism from shared pathway membership.

Psychological phenotypes require comparable precision. “Depression”, “anxiety”, and “stress” encompass differences in symptom domain, severity, chronicity, recurrence, treatment exposure, sleep, trauma, and social context, each of which may engage distinct biology. Immunometabolic abnormalities, e.g. characterize only a subset of people with depression [8]. Collapsing heterogeneous phenotypes into a single diagnostic label or symptom score may obscure opposing or null effects. PCIN studies should combine validated dimensional assessments with molecular, autonomic, imaging, behavioral, and environmental measurements, while rigorously addressing reverse causation, medication effects, socioeconomic adversity, physical inactivity, and other confounders. Genetic evidence suggesting only modest causal effects for depression liability on some cardiometabolic outcomes further argues against a single universal pathway [7].

For precision medicine, the decisive question is whether PCIN adds actionable information beyond established cardiovascular risk factors and standard psychiatric assessment. Prospective cohorts should repeatedly measure psychological dimensions, cardiovascular phenotypes, medication use, health behaviors, sleep and circadian patterns, social exposures, autonomic function, and a parsimonious set of molecular markers. Candidate biomarker panels should be evaluated for calibration, discrimination, reclassification, reproducibility, feasibility, and clinical utility—not merely statistical association [9]. External validation across sex, age, ancestry, socioeconomic context, psychiatric diagnosis, and CVD subtype will be essential. Multi-omics and machine learning may help define endotypes, but transparent models with prespecified validation are preferable to high-dimensional signatures that cannot be interpreted or reproduced.

Intervention studies provide the most stringent test of PCIN. Psychological therapies can improve depression and anxiety symptoms in coronary heart disease, heart failure, or atrial fibrillation, but their effects on cardiovascular events remain uncertain and heterogeneous [10]. Future trials should use PCIN-informed endotypes, measure candidate mediators before and during treatment, and assess both mental-health and cardiovascular outcomes. The molecular strategies discussed by Li and colleagues—including selective serotonin reuptake inhibitors, 5-HT_2_A-R antagonism, brain-penetrant NLRP3 inhibitors, TrkB agonism, and tissue-targeted gene delivery—are intriguing but remain unevenly supported and may produce tissue-specific benefits and harms [3]. The immediate value of PCIN is thus not a ready-made therapeutic algorithm, but a disciplined research agenda: define phenotypes more precisely, establish directional mechanisms, validate clinically useful biomarkers, and test whether integrated interventions improve outcomes in both organs. If those standards are met, then PCIN could help move brain–heart medicine from conceptual integration toward genuinely predictive and individualized care.

## Notes

**Editor’s note:** A commentary on “Psycho-cardiovascular interaction network in cardiovascular health and disease: signaling pathways and molecular mechanisms”
